# Diagnostic Challenges and Radiological Spectrum of Tumefactive Multiple Sclerosis: A Case Report Study

**DOI:** 10.7759/cureus.31899

**Published:** 2022-11-26

**Authors:** Rabia Muddassir, Sara B Badirah, Ghada M Alshahrani, Ayman A Eltahan

**Affiliations:** 1 Internal Medicine, Security Forces Hospital Makkah, Makkah, SAU; 2 Medicine, Umm Al-Qura University, Makkah, SAU; 3 Radiology, Security Forces Hospital, Makkah, SAU

**Keywords:** tumefactive demyelination, demyelinating disease, demyelination, multiple sclerosis, tumefactive

## Abstract

Multiple sclerosis (MS) is a chronic disease of the central nervous system (CNS). It has many types, which include tumefactive multiple sclerosis (TMS), one of the most uncommon types. We present the case of a 36-year-old woman who presented with right-sided numbness of the body. Magnetic resonance imaging (MRI) of the brain revealed a large mass (3 cm × 2.5 cm) in the deep white matter of the right frontal lobe along with smaller lesions of variable sizes. After considering the MRI features, the CSF results, and the improvement of the symptoms with a high dose of steroids, the diagnosis of tumefactive multiple sclerosis was made. A biopsy was not done on our patient as the symptoms resolved after treatment, although sometimes it is necessary for diagnosing tumefactive multiple sclerosis to rule out tumors or abscesses. The current study described the clinical presentation, the role of imaging, the differential diagnosis, and the treatment options. This case report aimed to report a rare presentation of TMS, which highlights the importance of differentiating TMS from other space-occupying lesions for prompt and proper management.

## Introduction

Multiple sclerosis (MS) is a chronic disease of the central nervous system (CNS). It is characterized by persistent demyelination, inflammation, and axonal injury, with recurrent attacks [1]. The prevalence of MS is two times more common in women than in men, and the onset of the disease is usually between 20 and 40 years old in 70% of patients [2]. Tumefactive multiple sclerosis (TMS) is a rare condition that has atypical imaging features that may mimic intracranial space-occupying lesions, typically more than 2 cm in diameter with a mass effect, edema, and ring enhancement [3]. The clinical presentation depends on the location of the lesion and its size; as a result, it has a wide variety of presentations such as headache, seizure, mental confusion, and cognitive abnormalities [4]. Nowadays, MRI, positron emission tomography (PET), and cerebral spinal fluid (CSF) analysis are used to make the diagnosis rather than invasive procedures like brain biopsy, which might pose considerable morbidity, particularly if it has the typical MRI features of TMS [1]. It has been estimated that there are three cases per million people per year affected by tumefactive MS, or 1-2 cases per 1000 cases of MS. Hence, the presentation of TMS may mimic brain tumors, cerebral abscesses, and inflammatory disorders, and it could be easily misdiagnosed [5]. The right frontal lobe mass and additional white matter lesions in our study were consistent with tumefactive multiple sclerosis. However, low-grade space-occupying lesions such as glioma, lymphoma, and stroke were included in the differential diagnosis. The current study aimed to report a rare presentation of TMS, which highlights the importance of differentiating TMS from other space-occupying lesions for prompt and proper management.

## Case presentation

**Figure 1:**
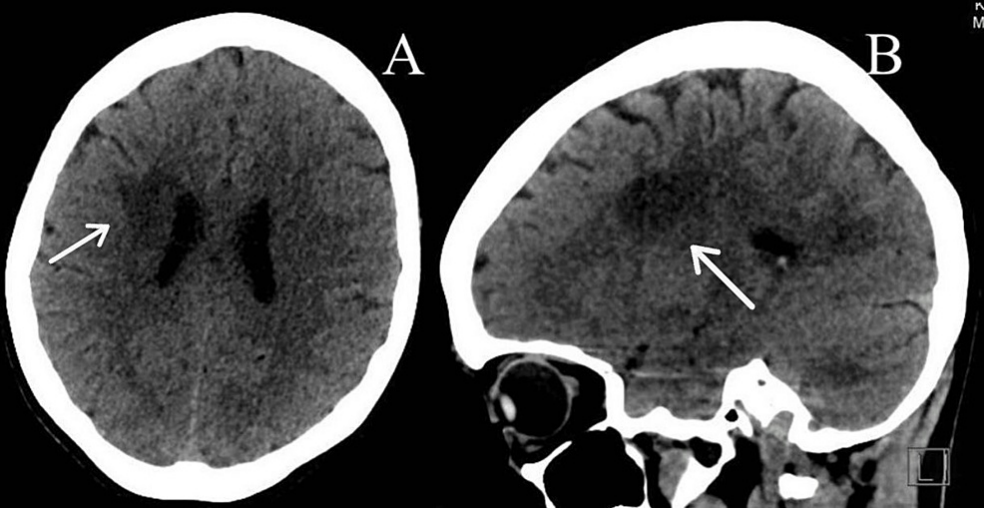
Brain CT (A) axial and (B) sagittal images show large right periventricular hypodensity.

A 36-year-old woman with no known history of chronic illness presented to the emergency department (ED) complaining of numbness in the right side of the body, followed by right lower limb weakness, which progressed until the patient walked aided by a cane. The symptoms began six days prior to the ED visit. There were no signs of ocular involvement, blurred vision, difficulty swallowing, difficulty speaking, headache, vertigo, or bladder incontinence. The patient’s medical history was remarkable for two similar attacks. The first attack involved left-sided weakness eight years ago; this attack was managed by herbal and traditional cautery. A brain MRI was done, but the patient did not receive the report. The second attack was two years ago and involved right-sided weakness with slurred speech and difficulty speaking, which improved spontaneously. During the initial neurological examination in the ED, the patient was conscious and oriented with intact higher mental functions, speech, and cranial nerves. Motor examination of both upper limbs showed normal tone; the power on the right side was 4/5 proximally as well as distally. Reflexes were normal. Motor exam of lower limbs showed normal tone with decreased power 4/5 proximally and distally. Reflexes were brisk. Sensory examination: superficial sensations were decreased in both the right upper and lower limbs; there were no dorsal column signs. The cerebellar exam was normal. Therefore, laboratory investigations were ordered. A brain computerized tomography (CT) scan showed a large right periventricular hypodensity with no significant mass effect on the surrounding structures, most likely an acute/subacute infarction (Figure 1).

**Figure 2:**
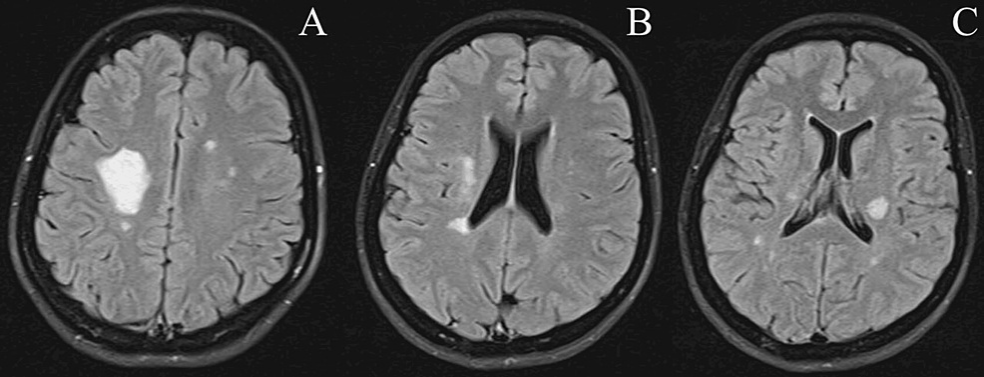
Brain MRI (A-B-C) axial FLAIR images show variable sized lesions in deep white matter of bilateral cerebral hemispheres with abnormal high signal intensity. The largest lesion in the right is 3 cm × 2.5 cm in size.

At this point, the patient was given Atorvastatin 40 mg, along with dual antiplatelet medications, and was referred to a neurologist for further evaluation. The next day, the examination by the neurologist was similar to the ED visit, except for preserved sensations. Laboratory investigations, including the complete blood count (CBC), coagulation profile, metabolic profile, and autoimmune profile, were all within the normal range. Rapid plasma reagin (RPR) was negative. The human immunodeficiency virus (HIV) test was negative. CSF results showed red blood cells (RBC) at 133 cells/µL, white blood cells (WBC) at 16 cells/µL, and protein at 33 mg/dL. CSF and serum oligoclonal bands were absent, and immunoglobulin G (IgG) was 67.7 mg/L (0.01-0.06 mg/L). She subsequently underwent a brain MRI, which showed variable-sized lesions in the deep white matter of bilateral cerebral hemispheres with abnormally high signal intensity in fluid-attenuated inversion recovery (FLAIR) (Figure 2).

**Figure 3:**
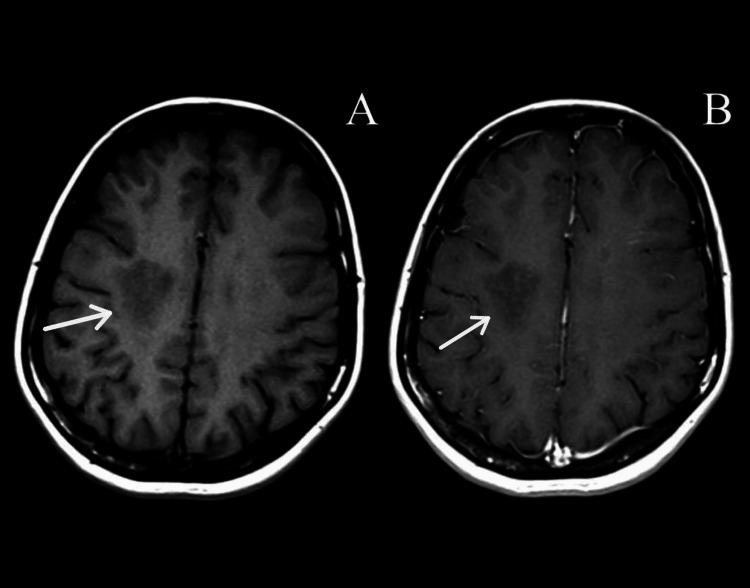
Brain MRI (A) axial T1 and (B) axial T1 post-contrast show no enhancement of the lesion in deep white matter of the right frontal lobe.

A brain MRI T1 post contrast showed no enhancement in the large right frontal lesion (Figure 3).

**Figure 4:**
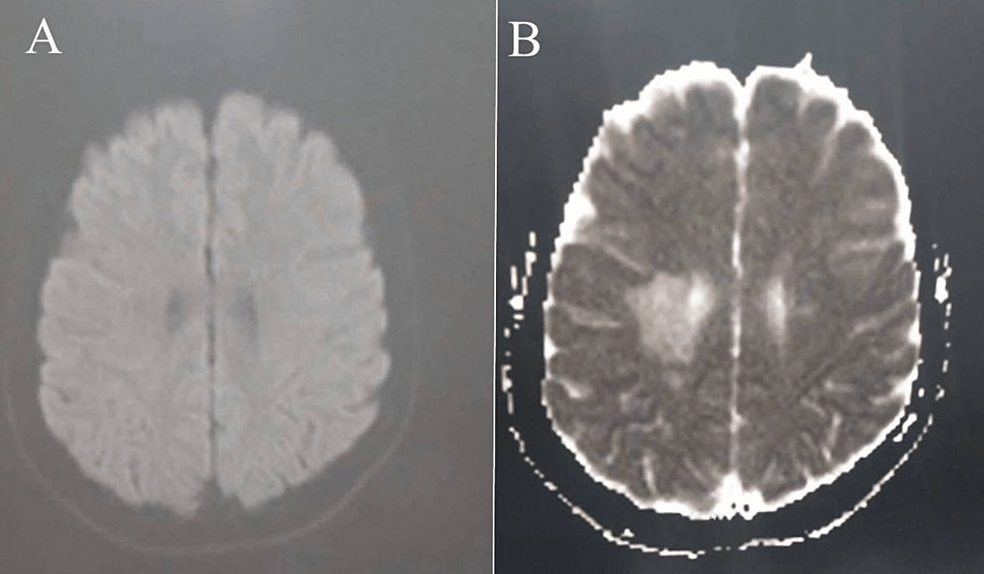
Brain Axial DWI/ADC MRI, show no restriction.

Diffusion-weighted imaging (DWI)/apparent diffusion coefficient (ADC) was done to exclude brain abscess which showed no restriction (Figure 4).

The imaging differentials thus considered were suggestive of multiple sclerosis, and low-grade space-occupying lesions such as gliomas and stroke. Low-grade glioma appears as a single lesion surrounded by perilesional edema, while in our study there are multiple lesions with no edema. The location of the lesions is atypical for stroke and suggestive of multiple sclerosis. A cervical MRI with contrast was done and showed no cervical cord plaques or abnormal signal intensities. The patient was admitted and given pulse steroid therapy for three days with the diagnosis of tumefactive multiple sclerosis. After the acute attack, the patient was started on fingolimod hcl 0.5 mg, and an appointment was arranged after one month at the end of 2018. The patient was lost to follow-up and came back to her appointment a year after the required date. She continued to take the prescribed medication after the hospitalization and had no attacks during that year. The MRI was repeated and showed no significant change to the size or new lesions. Through these three years up to date, the patient has been stable clinically and radiologically and has had no further attacks.

## Discussion

Multiple sclerosis affects different parts of the central nervous system, such as the brain, spinal cord, and optic nerve, causing a variety of neurological symptoms and disabilities. The plaques are generally found in the brainstem, cerebellum, periventricular, and juxtacortical white matter, as well as in the corpus callosum [6]. Multiple sclerosis has been classified into four disease courses: primary progressive MS (PPMS), secondary progressive MS (SPMS), relapsing-remitting MS (RRMS), and clinically isolated syndrome (CIS). The initial episode of neurologic symptoms is brought on by the inflammation and demyelination of the central nervous system, which is known as the CIS, and it must endure for at least 24 hours. In a certain group of people who have CIS, it may not develop into MS. Attacks of new or intensifying neurologic symptoms are features of RRMS. There are periods of partial or full recovery (remissions) following these attacks, which are also known as relapses or exacerbations. With no early relapses or remissions, PPMS is defined by a decline in neurological function (accumulation of disability) from the time that symptoms first appear. For many patients, SPMS is the stage that follows relapsing-remitting MS; in which neurologic function gradually deteriorates over time [7].

TMS is an uncommon variant of MS characterized by tumefactive demyelinating lesions (TDLs) larger than 2 cm and may be associated with mass effect and/or edema; it is often cannot be differentiated from CNS neoplasms, especially glioma and CNS lymphoma [3,6]. TMS is estimated to affect 1-3/1,000 MS cases, with an annual incidence of 0.3/100,000 people [8]. TDLs are not an uncommon manifestation of demyelinating disease, with an incidence of 2.8% of MS cases. In patients without a prior MS diagnosis, it might be a diagnostic challenge [4]. The typical sites of the lesions are focal and supratentorial, with the frontal and parietal lobes being mostly affected. The majority of MS patients present with motor, sensory, cognitive, or brainstem-related symptoms, and cerebellar involvement is also not uncommon. Due to the size and mass effect of the lesions, the clinical presentation of TMS may be unusual and more likely to result in symptoms such as memory loss, aphasia, and apraxia [6].

There are no pathognomonic characteristics in MRI for diagnosing tumefactive demyelinating lesions [6]. A study by Kim et al. suggested that MRI features that distinguish TDLs from gliomas or lymphomas are incomplete ring enhancement, mixed T2-weighted iso or hyperintensity of enhancing components, absence of mass effect, and cortical involvement. In lymphomas and gliomas, both T2-weighted and T1-weighted signal intensities of enhanced regions are variable. The variability in T1-weighted imaging seems to have an overlap in signal intensities between TDLs and tumors, which makes it unreliable for diagnosing TDLs. On the other hand, T2-weighted signal intensities in TDLs are iso or hyperintense, but variable in gliomas and lymphomas. Unenhanced CT and MRI together are more accurate than contrast-enhanced MRI alone in differentiating TDLs from gliomas or CNS lymphomas; in this report, both modalities have been used [9]. A non-contrast head CT is a valuable addition that could help distinguish tumefactive demyelinating lesions from tumors; the lesions that enhance in MRI appear hypodense on a non-contrast CT scan of the head. In tumefactive demyelination, this finding was present 93% of the time, compared to only 4% of the time in tumors [6].

A brain biopsy was done in 168 cases in a study by Lucchinetti et al., which demonstrated histopathological features of active inflammatory demyelinating disease, including hypercellularity with myelin loss, reactive gliosis, Creutzfeldt cells, myelin protein-laden macrophages, lymphocytic inflammation, and relative axonal preservation. In 31% of cases, demyelination was not the initial diagnosis. Low-grade astrocytoma was the most frequent misdiagnosis in 39% of cases, followed by high-grade astrocytoma [5]. A study reported that differentiating tumefactive multiple sclerosis and intracerebral lymphoma is difficult in immunocompromised patients, thus brain biopsy should be considered [10]. The literature has described different cases of TMS that have been diagnosed without brain biopsy depending only on radiological and CSF results; however, in some cases, brain biopsy was an essential procedure to confirm the diagnosis and avoid mismanagement [11]. In this current study, a brain biopsy was not performed.

Acute treatment of TMS is not specific; a high-dose intravenous corticosteroid was given to our patient as the typical first-line treatment. Patients who do not respond to steroids may benefit from plasma exchange, and the therapy may need to be escalated to rituximab or cyclophosphamide if there is no clinical improvement [6]. The time of recurrence was estimated to be 4.8 years, according to Lucchinetti et al. [5]. A study reported recurrence in five cases of TMS complicated by brain herniation that required an emergency decompressive craniotomy. In all the reports, the initial management included corticosteroids, and one osmotic diuretic, azathioprine (AZA), was added to the treatment in one study [12]. In most cases, a conservative approach with the use of radiological modalities and clinical follow-up is enough, while other studies used invasive surgical interventions such as brain biopsy or mass resection [13]. The clinical findings and the radiological features in our case were suggestive of tumefactive multiple sclerosis, as the patient had multiple attacks. Once the patient was given high-dose corticosteroids, her symptoms improved. Up to date, the patient is on fingolimod and symptom-free.

## Conclusions

TMS may mimic the diagnosis of other conditions, including ischemic stroke, low-grade space-occupying lesions, and MS. The present study provided a detailed description of the clinical presentation of each attack, the differential diagnosis, the diagnostic modalities used for the patient, and the available treatment options for tumefactive MS. Hence, TMS requires identification of its diverse, often unusual, and nonspecific clinical presentation and imaging findings for prompt diagnosis and optimum therapy.
